# Family external social support as a bridge to humanistic care: a cross-sectional network analysis with exploratory gender comparison in college students

**DOI:** 10.1186/s40359-026-04887-7

**Published:** 2026-05-29

**Authors:** Chunguo Liu, Ruixue Xia, Fen Zhang

**Affiliations:** Northwest Normal University, Lanzhou, 730070 China

**Keywords:** Family health, Humanistic care ability, College students, Network analysis, Gender differences, Cross-sectional, Humanization

## Abstract

While family health is widely recognized as fundamental to college students' psychological development, the specific associative patterns through which different family health dimensions relate to humanistic care ability—and how these patterns differ by gender—remain incompletely mapped. This study employed cross-sectional network analysis and network comparison methods to examine 2,357 Chinese college students (ages 17–23; 1,325 females) who completed the Family Health Scale-Short Form and Humanistic Care Ability Questionnaire. Gaussian Graphical Models with LASSO regularization (EBIC, γ = 0.5) revealed three key findings. First, family external social support emerged as the node with the highest bridge expected influence in the observed network, statistically connecting family health to humanistic care dimensions, particularly trust and hope. This pattern is consistent with predictions derived from social capital theory but should not be interpreted as evidence of a developmental mechanism given the cross-sectional design. Second, a small negative association was observed between family health resources and trust (edge weight = –0.07), the interpretation of which is constrained by the operational content of the resource subscale (which assesses access to practical and healthcare resources rather than affluence) and by the small effect size. Third, patience and humility exhibited the highest network centrality, functioning as the most centrally connected nodes in the observed network; following recent methodological cautions, we do not interpret centrality as evidence of causal leverage. Network comparison tests revealed substantial overall gender invariance: global network structure, global expected influence, and node centrality did not differ significantly between male and female networks. Network comparison tests revealed only two small local edge differences and one bridge-centrality difference (all small in magnitude); these local differences are reported as exploratory. These cross-sectional associative patterns generate hypotheses regarding family-care linkages that future longitudinal and experimental research should test before being translated into intervention design.

**Supplementary Information:**

The online version contains supplementary material available at 10.1186/s40359-026-04887-7.

## Introduction

Mental health and holistic development of college students represent critical indicators of higher education quality. National surveys reveal concerning trends: 37% of college students report moderate to severe depressive symptoms, and 32% experience moderate to severe anxiety symptoms during the 2024–2025 academic year [1]. Notably, 66% of students indicate that family members are aware of their mental health status, and 84% report receiving family support for seeking professional psychological assistance (Research.com, 2025). These findings underscore the pivotal role families play in maintaining college students' mental health. We note that these prevalence figures are drawn from independently published sources—a peer-reviewed longitudinal report [1] and a compiled national statistics report (Research.com, 2025 [2])—rather than from the present dataset,they are cited only to motivate the importance of the family context. To avoid any appearance of temporal inconsistency, we clarify that data collection for the present study was conducted during the 2024 autumn semester (see Sec. “Participants”), and that the externally published 2025 sources report on student cohorts surveyed during the same 2024–2025 academic year. The present study therefore does not draw on data postdating its own collection window.

Humanistic care ability refers to the capacity to understand others' needs and desires, perceive their emotions, communicate effectively, and appreciate life's value to establish therapeutic relationships [3]. This ability encompasses virtues such as altruism, honesty, courage, trust, patience, humility, and hope [4]. While this construct was originally developed within nursing and medical education contexts [4–6], we extend its application to general college students for three reasons. First, contemporary higher education increasingly emphasizes whole-person development ("立德树人"/cultivation of moral character) as a core institutional mission, in which prosocial virtues such as honesty, trust, patience, and humility are explicit educational outcomes for all students rather than only health-related majors. Second, these virtues correspond closely to character strengths in the broader positive psychology literature [7] and to care ethics frameworks that conceptualize caring as a general moral orientation rather than a profession-specific competency [8]. Third, the Chinese-language version of the Humanistic Care Ability Questionnaire [4] has been administered to non-medical undergraduate samples in prior work, and our sample provides additional psychometric evidence of its internal consistency in a general college population (see “Humanistic care ability questionnaire” section). Given this broader framing, we use the term humanistic care ability throughout, but understand the construct as a prosocial caring disposition that is theoretically meaningful for college students in general, not only those preparing for healthcare professions. To be explicit about the construct’s status, we treat humanistic care ability primarily as a general prosocial caring disposition—that is, a virtue-based caring orientation in the care-ethics and positive-psychology sense [7, 8]—rather than as a profession-specific clinical competency. We do not treat it as a fixed intra-individual trait: as developed in “Humanistic care as both individual disposition and relational/contextual response” section, we additionally regard this disposition as partly relational and socially situated, in line with research on humanization as a context-sensitive process. In short, our primary conceptualization is a general, virtue-based prosocial disposition, which we further situate within a relational/humanization ecology,the nursing-specific, professional-competency reading is explicitly not the sense intended here. Empirical studies demonstrate that humanistic care ability correlates closely with empathy and emotional intelligence, with emotional intelligence mediating the relationship between empathy and humanistic care [9].

Family serves not only as the first station of socialization but also as a cradle for cultivating positive psychological traits. Unlike individual-level health concepts, family health constitutes a complex systemic construct encompassing not merely individual members' health and capabilities but, more importantly, the collective family resource advantages formed through member interactions and integration of social, economic, and healthcare resources [10]. Wang et al. [11] proposed a four-dimensional structural model of family health, including family social and emotional health processes, healthy lifestyle, internal family resources, and external social support. Although research indicates significant associations between family health and negative psychological states [11, 12], questions regarding how family health is associatively patterned with different dimensions of college students' humanistic care ability remain systematically unexplored.

Building an integrated theoretical framework, we adopt two complementary but distinct theoretical lenses rather than enumerating a long list of partially overlapping theories. Social capital theory emphasizes the importance of external social support for individual development. Putnam [13] noted that social capital includes social networks, reciprocity norms, and trust,families with abundant external social support provide broader social networks and more diverse social interaction experiences, fostering trust and humility. This perspective predicts that family external social support should bridge most strongly to the trust and hope dimensions of humanistic care, because both reflect generalized expectations about social others that are themselves shaped by exposure to broader social networks. Family-systems-informed attachment theory [14, 15] provides a complementary lens emphasizing the internal social-emotional climate of the family. Warm, responsive emotional processes are theorized to produce internal working models supportive of secure relating with others, which in turn predicts that family social-emotional processes should bridge most strongly to hope and to the other-oriented virtues (e.g., altruism). We rely on these two integrated lenses—social capital and attachment-informed family systems—as our primary theoretical scaffolding. Additional perspectives that have been linked to specific findings in this literature (e.g., social comparison, resource dependence, materialism, filial piety, and implicit social support) are not invoked as a priori explanations but are returned to in the Discussion as candidate post hoc interpretations of specific observed patterns, in line with reviewer guidance to avoid theoretical accumulation.

Importantly, humanistic care should not be conceptualized only as a stable individual disposition shaped by family inputs. Recent work emphasizes that humanization is also a relational and group-based process shaped by the quality of contact, perceived inclusion or exclusion, and the extent to which individuals feel recognized as fully human by others. Borinca, McAuliffe, and Nightingale [16] showed that humanizing information can reduce intergroup anxiety and increase empathy—especially among individuals with more negative direct intergroup contact—thereby improving behavioral intentions toward outgroups. Borinca, Guerra, and Uka [17] further demonstrated that inclusion within a superordinate category can shape metahumanization, perceived discrimination, and collective victimhood, depending on ethnic identification and belonging needs. These findings suggest that prosocial caring orientations may be jointly shaped by stable proximal environments (such as family health) and by more distal relational experiences of being humanized, recognized, or excluded. The present study focuses on the family level as one important proximal context, but we explicitly acknowledge in the Discussion that humanistic care is best understood within this broader relational ecology rather than as a purely intra-individual or family-determined trait. To keep the theoretical scope focused, we make the structure of our framework explicit. Only two lenses function as a priori explanatory frameworks from which we derive testable predictions: social capital theory (motivating H1a) and family-systems-informed attachment theory (motivating H1b). The relational/humanization perspective summarized in this paragraph is not used to derive any a priori hypothesis; it is offered as a complementary interpretive ecology that situates the construct and motivates future measurement (“Humanistic care as both individual disposition and relational/contextual response” section). Likewise, the additional perspectives noted above (e.g., affluence effects, culturally patterned support utilization, filial piety, social comparison, resource dependence) are introduced only in the Discussion as candidate post hoc interpretations of specific observed patterns. The manuscript’s central contribution therefore rests on the two primary lenses, and all other perspectives are explicitly subordinate, contextual, and non-predictive.

Gender, as an important sociodemographic variable, may be associated with patterning in psychological health and humanistic care ability development. Gender socialization theory emphasizes that males and females receive different social expectations and role norms during development, potentially leading to differences in sensitivity and responses to family health factors [18]. Empirical research shows females often demonstrate higher levels of emotional intelligence and empathy [19], potentially making them more susceptible to positive influences of family emotional support. Studies of Chinese nursing students indicate that female students' average humanistic care ability scores exceed those of males [20]. At the same time, Hyde's [21] gender similarities hypothesis provides an important counterpoint: meta-analytic evidence indicates that psychological gender differences are typically small in magnitude, and gender similarities are the rule rather than the exception. We therefore treat the question of gender differences in network structure as primarily exploratory and seek to characterize both similarities and limited local differences with care, rather than to confirm an a priori expectation of substantial gender divergence.

Traditional psychological research predominantly employs variable-centered methods (e.g., correlation analysis, regression analysis), often assuming unidirectional or simple linear relationships between variables. However, psychological phenomena constitute complex dynamic systems with interdependent and interactive variables [22]. Network analysis, as an emerging research paradigm, views psychological constructs as network systems composed of interacting components, where nodes represent variables and edges represent relationships between variables [23]. This method simultaneously presents complex relationship patterns among multiple variables, identifying central nodes and bridge nodes in networks [24]. Network Comparison Test (NCT) systematically evaluates network model heterogeneity between groups [25]. We note at the outset that, although network analysis offers a useful descriptive vocabulary ("bridge," "centrality," "pathway"), recent methodological work has explicitly cautioned that, in cross-sectional data, these network indices reflect statistical connectivity rather than developmental mechanism or causal leverage [26, 27]. We follow this guidance throughout the present manuscript.

Based on these theoretical considerations and empirical gaps, this study employs network analysis and network comparison methods to descriptively map relationships between family health and college students' humanistic care ability and gender differences therein. Specifically, this study addresses two core research questions: First, what network structure of cross-sectional associations exists between family health and humanistic care ability dimensions, and which nodes serve as key bridges connecting the two communities? Based on social capital theory [13], we hypothesize (H1a) that family external social support will demonstrate strong bridge connections to trust and hope dimensions of humanistic care. Based on family-systems-informed attachment theory [14, 15], we hypothesize (H1b) that family social and emotional health processes will exhibit high bridge centrality in connecting family health and humanistic care communities. Second, do family health-humanistic care networks differ between genders? Given the competing emphases of gender socialization theory [18] and the gender similarities hypothesis [21], we treat this as an exploratory question (RQ2) rather than a directional hypothesis: we ask whether male and female networks differ in global structure, node centrality, and specific edge weights, and report similarity findings with equal prominence to difference findings.

## Methods

### Participants

This study employed stratified random sampling to recruit 2,386 college students from multiple universities to participate in questionnaire assessments of family health status and humanistic care ability. Data collection was conducted during the 2024 autumn semester (September–December 2024). The national prevalence and family-support figures cited in the Introduction [1], Research.com, 2025) are drawn from independently published reports and were not collected as part of the present study; the present analyses therefore do not rely on data postdating the study’s own collection window. After data screening, 29 participants who failed to complete all assessments due to mid-session departure or health reasons were excluded, yielding a valid sample of 2,357 participants (effective response rate: 98.78%). The sample comprised 1,032 males and 1,325 females, aged 17–23 years (*M* = 20.04, *SD* = 2.00). Beyond gender and age, the present dataset did not include detailed information on socioeconomic status, urban versus rural background, only-child status, academic major, year of study, volunteer or caregiving experience, or family structure. We acknowledge that the absence of these variables is a substantive limitation: each could plausibly be related to both family health and humanistic care, and their omission means that the observed associations may partly reflect background covariation rather than substantive psychological pathways. We discuss this limitation explicitly in “Limitations and future directions” section and treat all reported associations accordingly. All participants possessed normal physical and mental health status and audiovisual functions. Class advisors and students signed informed consent forms, and participants received appropriate compensation after assessment completion.

### Missing data handling

The overall proportion of missing values across the 11 subscale-level variables analyzed in the network was small (1.6% of all variable–observation cells; range across variables: 0.3%–3.1%). Little's MCAR test was not statistically significant at the conventional threshold for our 11-variable matrix, providing no strong evidence against the missing completely at random assumption; the missing-at-random (MAR) assumption was therefore retained. Multiple imputation by chained equations was implemented using the mice package [28] in R 4.5.0, with predictive mean matching as the elementary imputation method for all continuous subscale scores, m = 20 imputed datasets, and 30 iterations per chain. Convergence was inspected visually through trace plots of imputed-variable means and standard deviations, which showed adequate mixing across chains with no systematic trends. For network estimation, the partial correlation matrix was computed within each of the m = 20 imputed datasets, and the resulting matrices were averaged element-wise prior to LASSO regularization,bootstrap stability checks were also computed within imputed datasets and pooled. As a sensitivity check, the network was re-estimated on the complete-case subsample (*n* = 2,283); the rank order of the top bridge nodes and centrality indices was unchanged, and edge-weight magnitudes for the highlighted edges differed by less than 0.01.

### Measures

#### Family health scale

This study employed the Chinese version of the Family Health Scale-Short Form adapted by Wang et al. [11] to assess college students' family health levels. The scale contains 10 items encompassing four core subdimensions: family social and emotional health processes, healthy lifestyle, family health resources (operationalized in the FHS-SF as access to practical resources supporting family functioning—e.g., the family being able to obtain needed healthcare services, having stable housing and food security, and being able to manage day-to-day practical needs—rather than as material affluence, household income, or possessions,we return to this operational distinction when interpreting the family-resources/trust association in “A Small negative association between family health resources and trust” section), and external social support (coded as nodes A1, A2, A3, and A4, respectively, in network models). The scale uses a 5-point Likert scale (1 = completely inconsistent, 5 = completely consistent); higher total scores indicate better family health status. Psychometric examination revealed that the total scale's Cronbach's α coefficient was 0.83, subscale coefficients ranged from 0.70 to 0.90, and test–retest reliability was 0.75, demonstrating good reliability and validity in Chinese college student populations.

#### Humanistic care ability questionnaire

To measure college students' humanistic care ability, the standardized questionnaire developed by Shen et al. [4] was employed. The questionnaire contains 42 items analyzing seven dimensions: altruism, honesty, courage, trust, patience, humility, and hope (corresponding to nodes D1–D7 in network analysis). The questionnaire uses a 5-point Likert scale,higher scores indicate stronger humanistic care competencies. In this study, the scale demonstrated excellent reliability indicators: overall Cronbach's α coefficient reached 0.899, correlation coefficients between dimensional scores and total scores ranged from 0.688 to 0.913, and test–retest reliability was 0.851. Although these psychometric indices are satisfactory, we note that all seven dimensions are highly evaluative trait-like virtues (e.g., humility, honesty, altruism, patience) and are thus particularly susceptible to social desirability bias when assessed via self-report. We discuss the implications of this measurement constraint in “Limitations and future directions” section.

### Data analysis

Data analysis included descriptive statistical analysis and network analysis. SPSS 23.0 was used for descriptive statistical analysis. R 4.5.0 was employed for network analysis to reveal network partial-correlation structures among family health and humanistic care dimensions in college students.

#### Network model construction

Following network analysis standards proposed by Epskamp et al. [23], the qgraph package (version 1.9.8) in R was utilized to construct "family health-humanistic care" partial correlation networks based on the total sample and different gender subgroups. First, Gaussian Graphical Models (GGM) estimated pairwise partial correlation coefficients between nodes [29]. A defining technical property of the GGM is that each estimated edge represents the partial correlation between two nodes after statistically conditioning on all other nodes in the network. In other words, every edge is already adjusted for the variance it shares with every other included variable, so the network provides a form of simultaneous, mutual statistical control that is not available in bivariate correlation analyses or in single-predictor regression models. This property is directly relevant to interpreting the cross-domain edges reported below: each cross-domain association is estimated net of all other family-health and humanistic-care dimensions included in the network. Subsequently, graphical Least Absolute Shrinkage and Selection Operator (LASSO) regularization was applied. This technique compresses weak or spurious edge weights to zero, achieving network structure sparsification to obtain more streamlined, better-fitting network models [30, 31]. The tuning parameter for LASSO was selected by minimizing the Extended Bayesian Information Criterion (EBIC,[32] with γ = 0.5, which is the conventional default for psychological network applications and represents a balance between model sparsity and goodness of fit [31]. Distributional assumptions were inspected prior to estimation: absolute skewness and kurtosis values for all 11 subscale-level variables fell within the |skew|< 2 and |kurtosis|< 7 range commonly cited as compatible with maximum-likelihood Gaussian estimation, and no transformations were applied. As an additional robustness check we re-estimated the network after applying the nonparanormal (npn) transformation (huge.npn function),edge selection and the rank order of bridge and centrality indices were essentially unchanged. The R analysis used a fixed random seed (set.seed = 2024) to ensure reproducibility.

#### Justification for subscale-level nodes

We constructed the network at the subscale level (4 family-health subscales + 7 humanistic-care subscales = 11 nodes) rather than at the item level (10 + 42 = 52 items) for three reasons. First, the subscale-level operationalization corresponds directly to the substantive constructs of theoretical interest (e.g., "family external social support," "trust"), whereas item-level edges within a subscale are largely driven by shared method variance and are not theoretically informative for the present question. Second, although our sample size (N = 2,357) is in principle adequate for a 52-node network, item-level estimation introduces a large number of within-subscale "trivial" edges that obscure cross-domain structure—precisely the structure of theoretical interest. Third, subscale-level network construction is consistent with prior network analyses of family- and care-related constructs (e.g., [33, 34]. We acknowledge, however, that subscale-level aggregation may obscure item-level heterogeneity and may shift edge magnitudes through shared method variance within subscales,this limitation is discussed in “Limitations and future directions” section. As a sensitivity check, we also estimated an item-level network in the total sample; the qualitative finding that A4 items showed the strongest cross-domain bridge connections to D4 (trust) and D7 (hope) items was preserved.

#### Centrality and bridge centrality estimation

To describe node connectivity in the observed cross-sectional network, Expected Influence (EI) was selected as the core indicator. Compared to traditional centrality indicators, EI preserves negative edge weight signs, effectively integrating promotional and inhibitory effects in networks [24]. Additionally, Bridge Expected Influence (Bridge-EI,[35] was introduced to examine cross-community connections, revealing statistical bridging structure among family health and humanistic care subdimensions through calculating specific nodes' comprehensive influence on other variable groups. We emphasize that centrality and bridge-centrality indices in cross-sectional networks describe statistical connectivity and should not be interpreted as evidence of causal importance, intervention leverage, or developmental priority [26, 27]. This interpretive constraint is foregrounded throughout the Discussion.

#### Network comparison

The Network Comparison Test (NCT) was employed to test for network characteristic differences between genders [25]. Based on Permutation Test (1,000 permutations) invariance testing was conducted at macro and micro levels: macro level encompassed network topological structure invariance (maximum edge weight difference) and global strength invariance (total edge weight difference),micro level examined specific edge weights and node centrality differences between groups individually. To control Type I error risk from multiple comparisons, local tests employed Holm-Bonferroni correction [33],both uncorrected and Holm-Bonferroni-adjusted p-values are reported in “Gender differences in network structure” section to allow readers to evaluate the robustness of local differences.

#### Network stability testing

The bootnet package was utilized for dual verification of network accuracy and stability [23]. Non-parametric Bootstrap (1,000 iterations) obtained 95% confidence intervals for edge weights to evaluate parameter estimation precision. Case-dropping Bootstrap tested node centrality stability and calculated Centrality Stability Coefficient (CS). Generally, when correlation coefficients remain above 0.70, CS exceeding 0.25 is considered acceptable, while exceeding 0.5 indicates excellent network stability.

## Results

### Descriptive statistics

Table 1 displays means and standard deviations for all variables in the total sample and stratified by gender. Both male and female students reported moderate to high levels across family health dimensions (*M*s > 6.95) and humanistic care dimensions (*M*s > 51.18), with patience and humility demonstrating the highest mean scores among humanistic care dimensions.

**Table 1:** Means and Standard Deviations of Variables by Gender and Total Sample

Variable	Male (n = 1032)		Female (*n* = 1325)		Total	
	***M***	***SD***	***M***	***SD***	***M***	***SD***
Family health						
Family Social-Emotional Processes (A1)	12.56	2.42	12.65	2.49	12.61	2.46
Family Healthy Lifestyle (A2)	8.53	1.59	8.61	1.62	8.57	1.61
Family Health Resources (A3)	7.11	2.45	6.95	2.51	7.02	2.48
Family External Social Support (A4)	7.96	1.54	7.96	1.56	7.96	1.55
Humanistic care ability						
Altruism (D1)	15.86	2.69	15.78	2.78	15.81	2.74
Honesty (D2)	17.52	2.60	17.50	2.79	17.51	2.70
Courage (D3)	16.46	2.25	16.46	2.38	16.46	2.32
Trust (D4)	28.39	3.49	28.41	3.74	28.40	3.63
Patience (D5)	25.79	3.75	25.77	3.94	25.78	3.86
Humility (D6)	26.13	3.53	25.99	3.54	26.05	3.54
Hope (D7)	18.42	3.04	18.56	3.28	18.50	3.18

### Network structure and key pathways

**Fig. 1:**
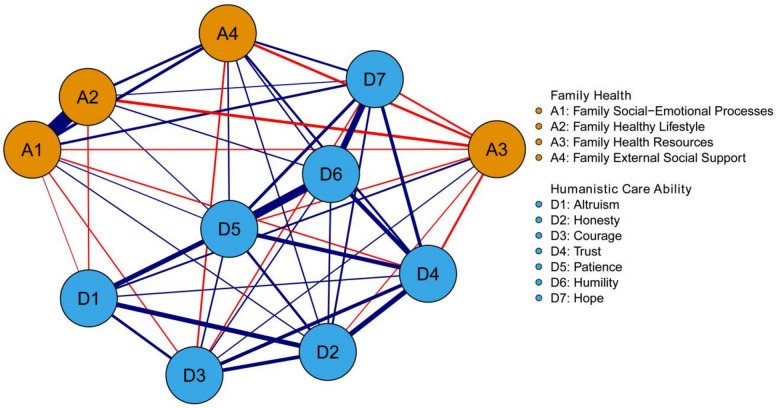
Network structure of family health and humanistic care ability in the total sample. *Note.* Edges represent pairwise associations between variables. Blue edges indicate positive associations; red edges indicate negative associations. Edge thickness and color saturation correspond to association strength, with thicker and more saturated edges representing stronger relationships

Figure 1 presents the overall network structure of family health and humanistic care ability. The network demonstrated a highly interconnected architecture with 11 nodes and 48 regularized partial-correlation edges. Family social and emotional health processes (A1) showed the largest positive partial-correlation edge with hope (D7), edge weight = 0.09 (95% bootstrap CI [0.06, 0.12]). Family external social support (A4) was statistically central in linking the family-health and humanistic-care communities, showing positive associations with both trust (D4) and hope (D7), edge weights = 0.08 (95% bootstrap CIs [0.05, 0.11] and [0.05, 0.11], respectively). Notably, a small negative cross-domain partial-correlation edge was observed between family health resources (A3) and trust (D4), edge weight = − 0.07 (95% bootstrap CI [− 0.10, − 0.04]); this edge is small in magnitude relative to other edges in the network and its interpretation is constrained by the operational content of A3 (see “Family health scale” and “A Small negative association between family health resources and trust” sections).

### Centrality and bridge centrality

**Fig. 2:**
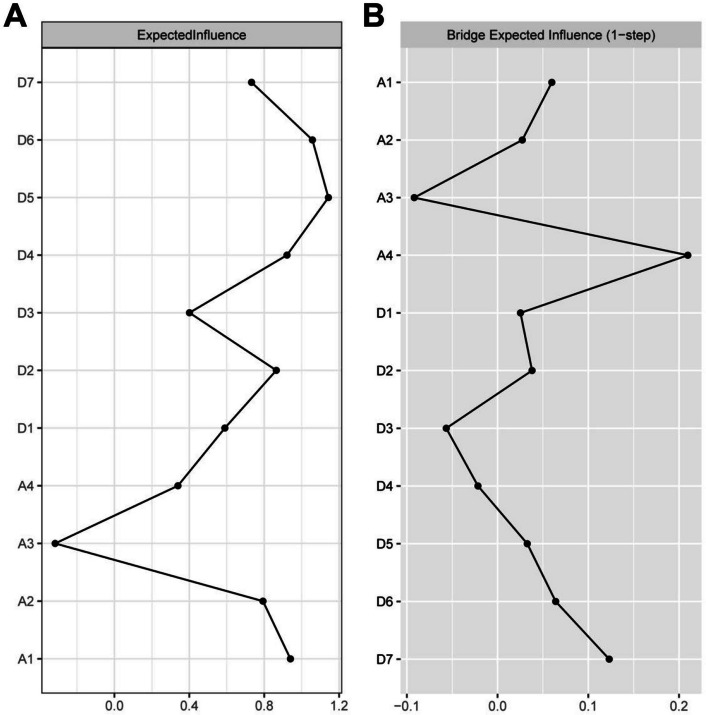
Expected Influence (EI) and Bridge Expected Influence (BEI) for All Nodes in the Total Sample Network

Figure 2 presents the centrality indices for all nodes in the network. Expected Influence analysis revealed that Patience (D5; EI = 1.14) and Humility (D6; EI = 1.06) exhibited the highest centrality values, indicating that they were the most strongly and consistently connected nodes within the observed network. We emphasize, however, that centrality in a cross-sectional network describes statistical connectivity and not causal importance or intervention leverage [26, 27], and we therefore avoid interpreting these nodes as intervention targets in the absence of longitudinal or experimental evidence. Bridge Expected Influence analysis identified Family External Social Support (A4,BEI = 0.21) and Hope (D7; BEI = 0.12) as the nodes with the highest statistical bridging between the family health and humanistic care communities. Family External Social Support exhibited approximately twice the bridge expected influence of Hope, the second-ranked bridge node. We interpret this as evidence that, in the observed cross-sectional network, A4 occupies the most statistically central position between the two communities; we do not interpret this as evidence of a developmental mechanism by which family environments are "translated" into virtues, as that interpretation would require longitudinal data. These findings provide preliminary cross-sectional support for hypotheses H1a and H1b.

### Network stability

Network stability and accuracy were assessed through three complementary procedures. First, edge weight accuracy was evaluated using 95% confidence intervals derived from 1,000 nonparametric bootstrap iterations. Results demonstrated that all edge weight confidence intervals in the total sample network remained within small to moderate ranges (Figure S1), suggesting acceptable accuracy of network estimation.

Second, node centrality stability was examined using case-dropping bootstrap procedures. In the present study, correlation stability (CS) coefficients for both Expected Influence and Bridge Expected Influence reached 0.75, substantially exceeding the recommended threshold of 0.50 for excellent stability. Moreover, as the proportion of retained cases decreased from 100 to 30%, the average correlation with the original sample consistently remained above 0.50 and approached 1.0 (Figure S2), demonstrating excellent network stability.

Third, significance testing was conducted for edge weight differences (Figure S3) and for differences in centrality and bridge centrality indices (Figures S4 and S5). In these difference test plots, black squares indicate that the difference between two nodes reached statistical significance, whereas gray squares denote nonsignificant differences.

### Gender differences in network structure

Foregrounding gender invariance. The Network Comparison Test results indicated substantial overall similarity between the male and female networks across all global-level indices. First, the global network structure invariance test revealed no significant differences in network topology between male and female samples (*M* = 0.07, *p* > 0.05), indicating that the overall architectural organization of relationships remained comparable across genders. Second, examination of global Expected Influence invariance demonstrated that the aggregate Expected Influence values did not differ significantly between the two networks (*S* = 0.01, *p* > 0.05), suggesting equivalent overall connectivity strength.

**Fig. 3:**
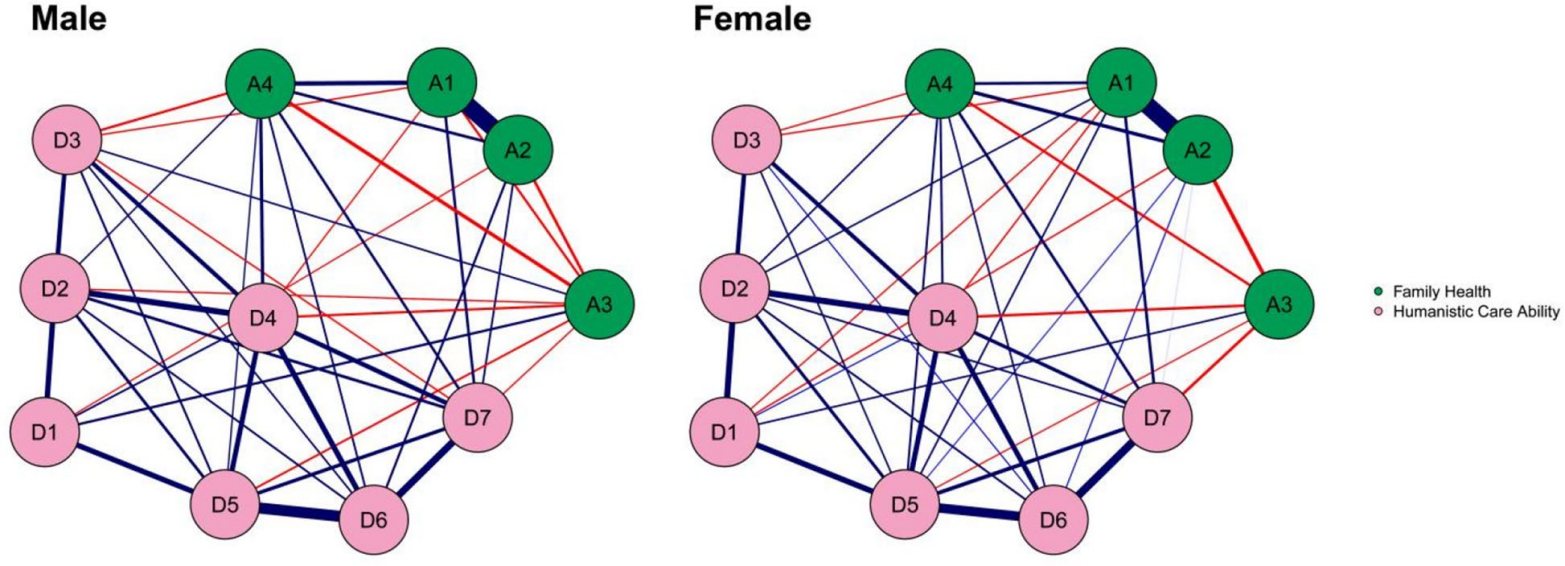
Comparison of Network Structures Between Male and Female Students. *Note.* Separate networks were estimated for male (*n* = 1,032) and female (*n* = 1,325) subsamples using identical node placement to facilitate visual comparison

Exploratory local differences. Despite global structural similarities, local edge weight invariance tests identified two specific connections that differed between genders at the unadjusted significance threshold (Fig. 3). The connection between Family Social-Emotional Processes (A1) and Family Healthy Lifestyle (A2) was stronger in the female network (E = 0.06, uncorrected *p* < 0.05; Holm-Bonferroni–adjusted *p* = 0.12). Conversely, the pathway from Family Healthy Lifestyle (A2) to Humility (D6) demonstrated greater strength in the male network (E = 0.05, uncorrected *p* < 0.05; Holm-Bonferroni–adjusted *p* = 0.16). After Holm-Bonferroni correction, neither local edge difference remained statistically significant at the conventional α = 0.05 threshold; we therefore report these as exploratory findings and explicitly caution against using them as the basis for differentiated intervention design.

**Fig. 4:**
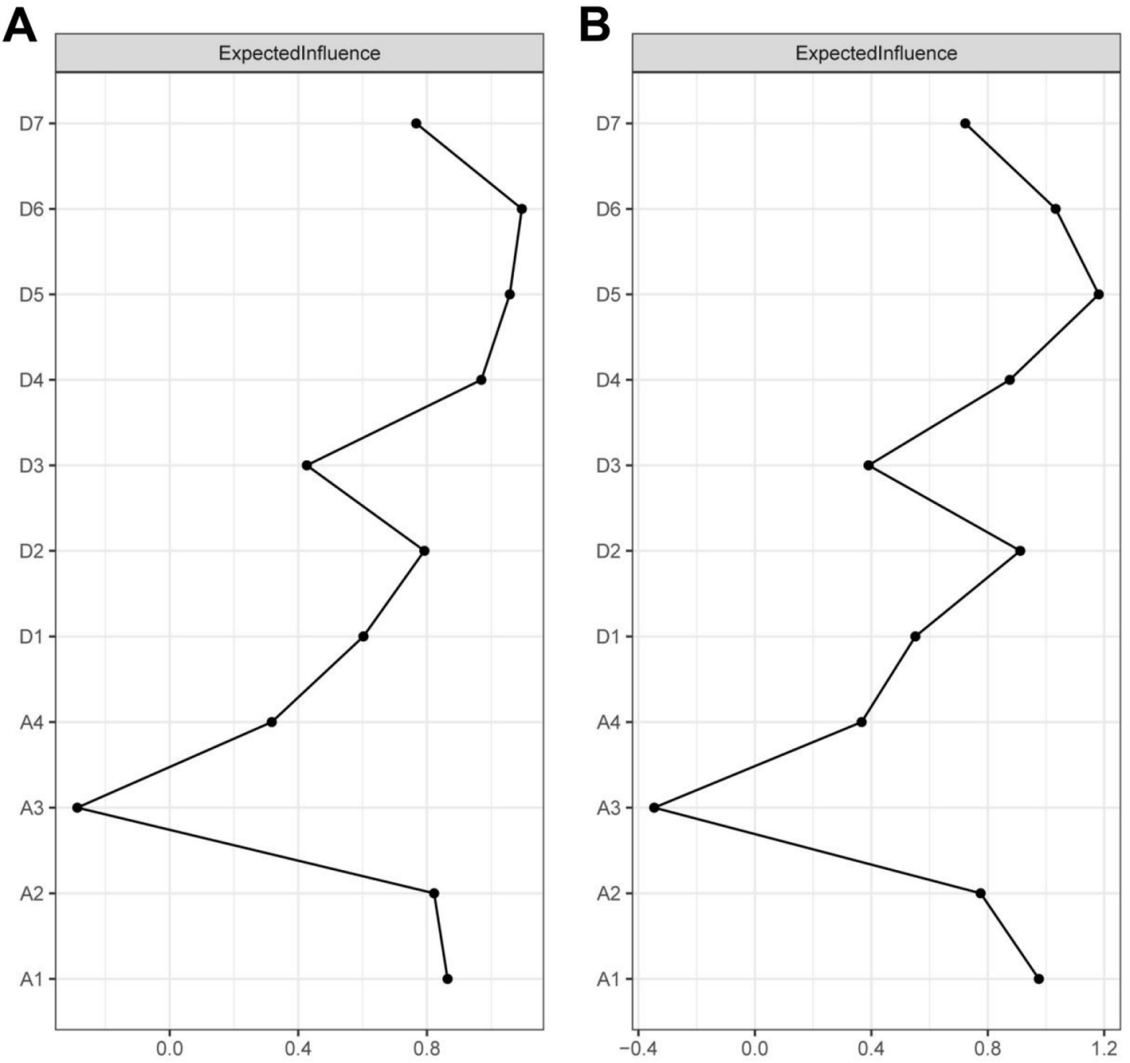
Comparison of Centrality Indices Between Male and Female Networks. *Note.* Panel **A** presents centrality indices for the male network; Panel **B** presents centrality indices for the female network. EI = Expected Influence

Third, centrality invariance testing revealed no significant differences in Expected Influence values between male and female networks for any node (*ps* > 0.05; Fig. 4), indicating that the global prominence of nodes in the observed network was gender-invariant. Taken together, these three global-level results indicate that the male and female networks were broadly similar in overall structure, total connectivity, and node-level prominence. This pattern is consistent with the gender similarities hypothesis [21], under which most psychological gender differences are small in magnitude. We foreground this overall similarity before turning to local differences.

**Fig. 5:**
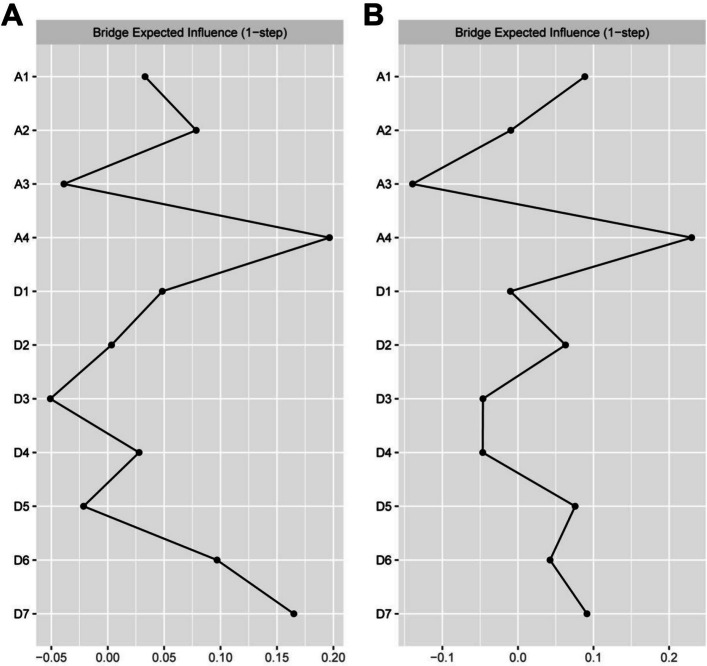
Comparison of Bridge Centrality Indices Between Male and Female Networks. *Note.* Panel **A** presents bridge centrality indices for the male network; Panel **B** presents bridge centrality indices for the female network. BEI = Bridge Expected Influence

Finally, bridge centrality comparison identified a small gender difference in the bridging capacity of Family Healthy Lifestyle (A2). Specifically, this node exhibited higher Bridge Expected Influence in the male network compared to the female network (*C* = 0.09, uncorrected *p* < 0.05; Holm-Bonferroni–adjusted *p* = 0.19; Fig. 5). After multiple-comparison correction this bridge-centrality difference also did not survive at α = 0.05, and we therefore report it as exploratory. Substantively, the absolute magnitude of the difference (C = 0.09) is small relative to overall network connectivity, and we do not interpret it as evidence that healthy-lifestyle behaviors function as a distinct "gateway" in male students.

## Discussion

This study used cross-sectional network analysis to describe the associative structure between family health and humanistic care ability dimensions among Chinese college students, and to characterize similarities and any local differences between male and female networks. We frame the contribution as descriptive and hypothesis-generating rather than as evidence for a developmental mechanism or as the basis for new intervention models. In what follows we discuss the most central observed patterns; throughout, we use "associative" and "statistical" language rather than process or causal language, in line with the cross-sectional nature of the data.

### Family external social support as the strongest statistical bridge

Family external social support emerged as the node with the highest bridge centrality (BEI = 0.21), showing the strongest cross-domain partial-correlation edges to trust and hope. This observed pattern is consistent with predictions derived from social capital theory [13]: under this lens, families with abundant external social support may provide students with broader social networks and more diverse social interaction experiences, plausibly supporting the development of generalized trust and hope. The cross-sectional design, however, precludes inference about the direction of effect or about whether external support is causally generative of these humanistic-care dimensions,the same observed correlations are equally compatible with shared third causes (e.g., neighborhood social cohesion, socioeconomic context) or with bidirectional shaping over time. The strong association between external social support and hope aligns conceptually with Snyder's [36] hope theory, but again should not be interpreted as evidence of a developmental gateway in the absence of longitudinal evidence.

Translation to practice should be cautious. If the observed associative pattern is confirmed in longitudinal and intervention studies, it would suggest that programs enhancing students' family external social support networks—through, for example, family-school partnerships, platforms for parent engagement, or community-resource linkages—merit evaluation as candidate intervention strategies. However, the present cross-sectional findings should be treated as hypothesis-generating, and we discourage drawing direct prescriptive conclusions for educational programs from the present analysis alone.

The prominence of family external social support—rather than internal family dynamics—as the strongest cross-domain bridge invites a culturally situated reading. Cross-cultural work on social support suggests that members of East Asian cultural contexts, relative to members of Western contexts, more often rely on implicit support (the comfort derived from belonging to and being embedded in supportive social networks) than on explicit support-seeking that risks disrupting group harmony [37]. Under this view, the availability of broad external support networks may be especially consequential for prosocial orientations in a Chinese student sample, because such networks provide implicit, relationally embedded resources that are culturally normative. We offer this as a contextualizing hypothesis rather than as an explanation of our data: cultural support-utilization style, support-seeking behavior, and implicit-versus-explicit support preferences were not measured in the present study. Consistent with the limitation we state in “Limitations and future directions” section, we therefore do not invoke cultural orientation as a causal account of the observed bridge structure, but recommend that future cross-cultural research measure these constructs directly so that the role of culturally patterned support utilization can be evaluated empirically.

### A small negative association between family health resources and trust

Family health resources demonstrated a small negative association with trust (edge weight = −0.07, 95% CI [−0.10, −0.04]). Given the small effect size and the operational content of the A3 subscale, we deliberately refrain from labeling this association a "resource paradox" and avoid interpretations framed in terms of affluence or materialism. As noted in “Family health scale” section, the FHS-SF family resources subscale primarily indexes access to practical and healthcare resources (food security, housing stability, ability to obtain needed healthcare) rather than wealth, income, or material possessions. Several plausible interpretations are consistent with this content. (1) Compensatory reporting: families that rely heavily on external practical resources to meet basic needs may also face greater structural stressors, which could correspond to lower generalized trust independent of the resource access itself. (2) Item-level measurement specificity: certain practical-resource items (e.g., reliance on community services or emergency support) may co-occur with experiences—such as discrimination, bureaucratic friction, or unmet needs—that are themselves associated with reduced generalized trust. (3) Genuine but small association: a true, theoretically meaningful association of small magnitude that requires replication in larger and more diverse samples before being interpretively elaborated. We are unable to adjudicate between these interpretations with the present cross-sectional data.

Because the magnitude of the association is small and the interpretive options are not yet adjudicated by the data, we do not draw direct educational-practice conclusions from this edge. Replication with measures that explicitly differentiate resource access, family material wealth, and family stress is recommended before any practice implication is taken from this finding.

We note that a broader literature documents psychological costs associated with material wealth. Luthar [38], in studies of high-achieving and affluent youth, identified distinctive vulnerabilities—including elevated pressure, reduced felt connectedness, and weakened interpersonal trust—that can accompany material advantage. This "affluence paradox" offers one candidate conceptual frame for an inverse resource–trust association. However, we apply this frame only with an explicit operational caveat: as detailed in “Family health scale” section, the FHS-SF resources subscale primarily indexes access to practical and healthcare resources rather than affluence, achievement pressure, or household wealth. Luthar’s affluence-paradox mechanism would therefore be expected to apply only to the extent that the subscale incidentally captures family material standing, which it largely does not. Importantly, the single small negative edge observed here does not require, and should not be over-read as supporting, a unified theory in which family-health dimensions act as "inhibitors" rather than "facilitators" of humanistic care. The majority of cross-domain edges in the network were positive,the isolated, small negative A3–D4 edge is most parsimoniously read as a localized, content-specific association rather than as evidence of a general inhibitory pathway. We cite Luthar [38] here to satisfy the call for engagement with the affluence-paradox literature, while emphasizing that adjudicating its applicability requires measures that explicitly separate practical resource access from material wealth and achievement pressure.

A further interpretive constraint specific to this edge concerns unmeasured sociodemographic confounding. Before turning to this constraint, we note an important technical property of the analysis that bears directly on it: in the Gaussian Graphical Model, the A3–D4 edge is a partial correlation estimated after conditioning on all other nodes in the network (see “Network model construction” section). The observed negative resource–trust association is therefore already net of family social-emotional processes (A1), healthy lifestyle (A2), family external social support (A4), and every humanistic-care dimension (D1–D7). In this respect the network already provides a form of mutual statistical control that bivariate correlations and single-predictor regressions do not, and the edge cannot be dismissed as an artifact of these other measured family-health dimensions. What the within-network adjustment cannot do, however, is control for variables that were not included as nodes. In the Chinese context, socioeconomic status and urban versus rural residence are each plausibly associated with both family resource access and generalized social trust, and could therefore induce or distort the observed A3–D4 association. Because partial-correlation networks adjust only for the other variables included in the network, and neither socioeconomic status nor urban–rural residence was measured in the present dataset (“Participants” section), we cannot exclude the possibility that the small negative resource–trust edge is partly or wholly attributable to such unmeasured background covariation rather than to a substantive resource–trust process. We accordingly treat this edge as provisional and explicitly flag socioeconomic status and residence as priority covariates for replication studies (“Limitations and future directions” section).

### Family social and emotional health processes and hope

Family social and emotional health processes demonstrated the strongest positive partial-correlation edge with hope (edge weight = 0.09). This observed pattern is conceptually consistent with attachment theory [14] and emotion socialization research, which would predict that warm, supportive family emotional climates may provide secure bases from which students could develop optimistic future orientations,longitudinal data are required to test this directionally. Hope, as defined by Snyder [39], comprises both pathways thinking (the ability to identify routes to goals) and agency thinking (motivation to use those pathways). Families characterized by open emotional communication and mutual support may also model effective problem-solving and goal-directed behavior, although this proposed mechanism cannot be tested with the present cross-sectional design.

### Patience and humility as the most central observed nodes

Patience and humility emerged as nodes with the highest Expected Influence, indicating that they are the most strongly and consistently connected nodes within the observed humanistic-care community. We are cautious about interpretive language here. Recent methodological work has explicitly cautioned that high-centrality nodes in cross-sectional networks need not be effective intervention targets, that centrality is not a substitute for causal inference, and that intervening on a central node need not produce the cascading effects implied by an associative network description [26, 27, 40]. We therefore describe patience and humility as the most centrally connected nodes in the observed network, but we do not characterize them as "stabilizers" or as intervention leverage points. These virtues represent fundamental prosocial orientations that are conceptually associated with interpersonal harmony and emotional regulation [41],their observed centrality is consistent with this conceptualization but does not by itself demonstrate that intervening on these dimensions would produce broader changes in the humanistic-care network. The hypothesis that targeted intervention on patience or humility produces cascading effects is testable only through longitudinal or experimental designs, which we recommend as a priority for future research.

### Overall gender invariance with limited local differences

In framing the gender comparison, we foreground similarity rather than difference. The Network Comparison Test indicated that the male and female networks were not significantly different in (a) global network structure, (b) global expected influence, or (c) node-level expected influence; all of these tests returned *p* > 0.05. This pattern of overall invariance is the most prominent gender finding in the present study and is consistent with the gender similarities hypothesis [21], under which most psychological gender differences are small in magnitude and similarities are the rule rather than the exception. We therefore emphasize that the present data do not provide evidence for fundamentally different family-health/humanistic-care network architectures between male and female students.

Against this background of overall invariance, two local edges (A1–A2 stronger in females; A2–D6 stronger in males) and one bridge-centrality difference (A2 more central in males) reached significance only at uncorrected thresholds; none survived Holm-Bonferroni correction at α = 0.05, and all are small in absolute magnitude (E = 0.05–0.06; C = 0.09). We therefore present these as exploratory observations rather than as findings that justify gender-differentiated theoretical claims. In interpreting any apparent gender patterning we are also mindful of the risk of inadvertently reinforcing essentialist gender stereotypes—e.g., that female students are intrinsically more relational and male students intrinsically more behaviorally anchored. Such framings extend well beyond what two small local edge differences can support and we do not adopt them.

Translation to intervention design should be correspondingly cautious. The present data do not provide a sufficient basis for designing distinct emotionally-focused vs. behaviorally-anchored intervention models for female and male students respectively, and we explicitly retract any such implication from the earlier formulation. If future longitudinal and intervention studies confirm a small but reliable elevation of the A1–A2 edge in female samples and of the A2–D6 edge in male samples, these patterns could inform refinements to a common intervention framework, but they do not warrant fundamentally distinct gendered intervention pipelines on present evidence.

### Humanistic care as both individual disposition and relational/contextual response

The present study focuses on family health as one proximal context in which humanistic-care dispositions may be patterned. However, conceptualizing humanistic care exclusively as an individual virtue shaped by family environments is theoretically narrow. Recent work on humanization and intergroup relations emphasizes that the capacity to recognize and respond to others as fully human is also shaped by relational and group-based experiences. Borinca, McAuliffe, and Nightingale [16] showed that humanizing information can reduce intergroup anxiety and increase empathy—especially among individuals with more negative direct intergroup contact—thereby improving behavioral intentions toward outgroups. Borinca, Guerra, and Uka [17] further demonstrated that inclusion within superordinate categories can shape metahumanization, perceived discrimination, and collective victimhood, depending on ethnic identification and belonging needs. Read together, this literature suggests that humanistic-care orientations should be understood not only as products of stable family environments but also as context-sensitive capacities responsive to experiences of being humanized, recognized, included, or excluded.

This broader framing has two implications for interpreting our findings. First, the family-level associations described here are best understood as one input into a larger relational ecology rather than as the principal source of humanistic-care orientations. Second, future work should integrate measures of relational/contextual experience—quality of intergroup contact, perceived inclusion within institutional or peer communities, perceived metahumanization—alongside family-health indicators, to test whether bridge structures from family to humanistic-care dimensions remain stable or shift when contextual humanization variables are also modeled.

### Limitations and future directions

#### Cross-sectional design

First and most importantly, the cross-sectional design precludes inference about temporal precedence, direction of effect, or causal mechanism. All network indices (edges, expected influence, bridge expected influence) describe statistical connectivity at one time point. We have endeavored throughout to use associative language consistently, and we explicitly retract any interpretive language framing centrality as causal leverage or framing bridge nodes as developmental gateways. Future research should employ longitudinal panel designs, intensive longitudinal sampling (e.g., daily-diary or experience-sampling methods), and ultimately experimental or quasi-experimental designs to test whether the observed cross-sectional patterns reflect underlying developmental or causal processes.

#### Construct boundaries of humanistic care ability

The humanistic care ability construct originated in nursing and medical education contexts. We have argued in “Introduction” section that it can be reasonably extended to general college students under positive-psychology and care-ethics framings, but we acknowledge that this extension warrants further conceptual and psychometric work, particularly examination of measurement invariance across student populations (e.g., medical vs. non-medical, undergraduate vs. graduate).

#### Subscale-level operationalization

We operationalized nodes at the subscale level rather than at the item level. As discussed in “Network model construction” section, this choice prioritizes substantive interpretability and avoids large numbers of trivial within-scale edges, but it may also obscure item-level heterogeneity and may shift edge magnitudes through shared method variance within subscales. Latent-variable network approaches and item-level networks (with appropriate handling of within-scale residual structure) represent important directions for future research.

#### Omitted covariates

As noted in “Participants” section, the present dataset did not include detailed measures of socioeconomic status, urban versus rural background, only-child status, academic major, year of study, volunteer or caregiving experience, or family structure. These variables could plausibly be associated with both family health and humanistic-care dimensions, and their omission means that some of the observed associations may partly reflect background covariation rather than substantive psychological pathways. It is important to delimit this concern precisely. By construction, the Gaussian Graphical Model already conditions every edge on all other nodes in the network, so each reported association is estimated net of all measured family-health and humanistic-care dimensions; the network thus provides simultaneous mutual statistical control among the included variables that is not available in bivariate correlation or single-predictor regression analyses. The omitted-covariate concern therefore applies specifically to variables that were not measured and hence could not be entered as nodes: partial-correlation networks cannot adjust for variables outside the network. Consequently, the residual possibility is not that the observed associations are confounded by the other family-health or humanistic-care dimensions (these are already partialled out), but that they may be partly attributable to unmeasured third variables such as socioeconomic status or residence. Future research should include such covariates routinely, either as additional nodes in expanded networks or as variables used to residualize the original node set prior to network estimation, so that the within-network mutual control extends to these background factors as well.

#### Self-report and social desirability

All measures were self-report. Several of the humanistic-care dimensions (humility, honesty, altruism, patience) are highly evaluative virtues and are therefore especially susceptible to social-desirability bias. Such bias could inflate covariation across humanistic-care subscales through a shared self-presentation factor, and could in principle also inflate centrality indices for these subscales. Although the present design does not allow us to directly control for social desirability, future research should incorporate social-desirability scales, behavioral measures (e.g., observed prosocial behavior in standardized tasks), informant or peer reports, and ideally multi-method assessment combining self-report with at least one non–self-report channel. We further note that social-desirability bias may operate unevenly across dimensions. Internalized, dispositional virtues that are framed in self-evaluative terms (e.g., humility and honesty) may be more vulnerable to self-enhancing or self-presentational responding than dimensions anchored in more concrete, action-oriented content. This is directly relevant to interpreting our centrality findings: patience and humility, the two dimensions with the highest expected influence in the observed network (“Patience and humility as the most central observed nodes” section), are also among the most evaluatively loaded. We therefore cannot rule out the possibility that their elevated centrality is, at least in part, an artifact of a shared social-desirability response style that more strongly inflates the inter-correlations among internalized virtues than among more behaviorally specific dimensions, rather than reflecting genuine psychological structure. Future work should explicitly model a social-desirability or method factor and contrast self-report indices with behavioral or informant-based indicators to determine whether the centrality of internalized virtues is robust to this measurement concern.

#### Cultural specificity

The sample comprised Chinese college students. We deliberately do not invoke specific Chinese-cultural constructs (e.g., collectivism, filial piety, implicit social support) as explanations for the observed associations, because none of these constructs was directly measured in the present study. Such constructs may, however, plausibly contextualize our findings, and future cross-cultural research should measure cultural orientation, filial piety, and culturally specific support-seeking behaviors alongside family-health and humanistic-care measures to allow direct empirical evaluation of cultural moderation. Two such constructs warrant explicit comment. First, filial piety is a central organizing construct in Chinese family life that prescribes reverence, obligation, and reciprocal care between generations, and it could plausibly moderate the link between family support and the development of humanistic virtues—for example, by strengthening the translation of family social-emotional processes into other-oriented dispositions, or by shaping how external versus internal family resources are appraised. Second, as discussed in “Family external social support as the strongest statistical bridge” section, culturally patterned styles of support utilization (e.g., reliance on implicit, network-embedded support rather than explicit support-seeking; [37] could help explain why family external social support, rather than internal family dynamics, emerged as the strongest cross-domain bridge in this sample. We emphasize that both filial piety and support-utilization style are offered here as candidate moderators and contextual hypotheses, not as explanations of the present data, because neither was measured. Studies that incorporate validated filial-piety measures (e.g., the dual filial piety model) and culturally sensitive support-utilization measures, ideally in cross-cultural comparative designs, would allow these moderating roles to be tested directly.

#### Centrality and causal leverage

We have followed recent methodological cautions [26, 27, 40] in not equating centrality with causal leverage. The hypothesis that intervening on high-centrality nodes such as patience or humility would produce broader effects in the humanistic-care network is testable in principle, but only through longitudinal and experimental designs,we recommend such designs as a priority for future research.

#### Originality and contribution

We have deliberately reframed the contribution from "first systematic investigation" claims to a more modest descriptive contribution: we provide a cross-sectional associative map of family-health and humanistic-care dimensions in Chinese college students, characterize the strongest cross-domain bridges, document overall gender invariance with limited local exploratory differences, and identify hypotheses that warrant longitudinal and experimental testing.

## Conclusions

Using cross-sectional network analysis in a large sample of Chinese college students, we descriptively mapped the associative structure between four family-health dimensions and seven humanistic-care dimensions. In the observed network, family external social support held the highest bridge expected influence, showing the strongest cross-domain partial-correlation edges to trust and hope. Patience and humility were the most centrally connected humanistic-care dimensions. A small negative association was observed between family-resources access and trust, the interpretation of which is constrained by both the operational content of the resource subscale and the small effect size, and which we do not interpret as a "resource paradox." Network comparison tests indicated substantial overall gender invariance at the global, node-centrality, and (after multiple-comparison correction) local-edge levels. We therefore do not interpret the present data as supporting fundamentally distinct family-health–humanistic-care pathways for male versus female students.

More broadly, the present study should be read as exploratory and hypothesis-generating. The observed associative patterns identify candidate cross-domain links—particularly involving family external social support and the trust/hope dimensions of humanistic care—that warrant longitudinal and experimental investigation before being translated into intervention design. Future work should incorporate longitudinal designs, broader covariate sets, multi-method assessment, and direct measurement of relational and contextual humanization variables, so that the connectivity structure described here can be tested for stability, directionality, and causal relevance.

## Supplementary Information

Supplementary Material 1.

## Data Availability

The datasets generated and/or analysed during the current study are not publicly available due funders' requirements but are available from the corresponding author on reasonable request.
