# Supplementary material for: Family external social support as a bridge to humanistic care: a cross-sectional network analysis with exploratory gender comparison in college students
Source: BMC Psychol. 2026 May 29;14:1112. doi: 10.1186/s40359-026-04887-7 (PMC13412319; doi:10.1186/s40359-026-04887-7)
Supplement: Supplementary file 1 — Supplementary Material 1. [file 40359_2026_4887_MOESM1_ESM.docx]

**Supplementary Materials**

**Table S1**

Edge Weight Matrix for Family Health and Humanistic Care Ability in College Students

|  | **A1** | **A2** | **A3** | **A4** | **D1** | **D2** | **D3** | **D4** | **D5** | **D6** | **D7** |
| --- | --- | --- | --- | --- | --- | --- | --- | --- | --- | --- | --- |
| **A1** | 0.00 | 0.78 | -0.02 | 0.12 | -0.01 | 0.02 | -0.02 | -0.03 | 0.01 | 0.00 | 0.09 |
| **A2** | 0.78 | 0.00 | -0.11 | 0.10 | -0.03 | 0.00 | 0.00 | 0.00 | 0.01 | 0.03 | 0.01 |
| **A3** | -0.02 | -0.11 | 0.00 | -0.09 | 0.06 | -0.01 | 0.02 | -0.07 | -0.03 | 0.00 | -0.05 |
| **A4** | 0.12 | 0.10 | -0.09 | 0.00 | 0.00 | 0.03 | -0.05 | 0.08 | 0.04 | 0.04 | 0.08 |
| **D1** | -0.01 | -0.03 | 0.06 | 0.00 | 0.00 | 0.23 | 0.10 | 0.03 | 0.23 | -0.02 | 0.00 |
| **D2** | 0.02 | 0.00 | -0.01 | 0.03 | 0.23 | 0.00 | 0.16 | 0.24 | 0.09 | 0.04 | 0.06 |
| **D3** | -0.02 | 0.00 | 0.02 | -0.05 | 0.10 | 0.16 | 0.00 | 0.15 | 0.04 | 0.03 | -0.03 |
| **D4** | -0.03 | 0.00 | -0.07 | 0.08 | 0.03 | 0.24 | 0.15 | 0.00 | 0.19 | 0.20 | 0.14 |
| **D5** | 0.01 | 0.01 | -0.03 | 0.04 | 0.23 | 0.09 | 0.04 | 0.19 | 0.00 | 0.43 | 0.12 |
| **D6** | 0.00 | 0.03 | 0.00 | 0.04 | -0.02 | 0.04 | 0.03 | 0.20 | 0.43 | 0.00 | 0.31 |
| **D7** | 0.09 | 0.01 | -0.05 | 0.08 | 0.00 | 0.06 | -0.03 | 0.14 | 0.12 | 0.31 | 0.00 |

**
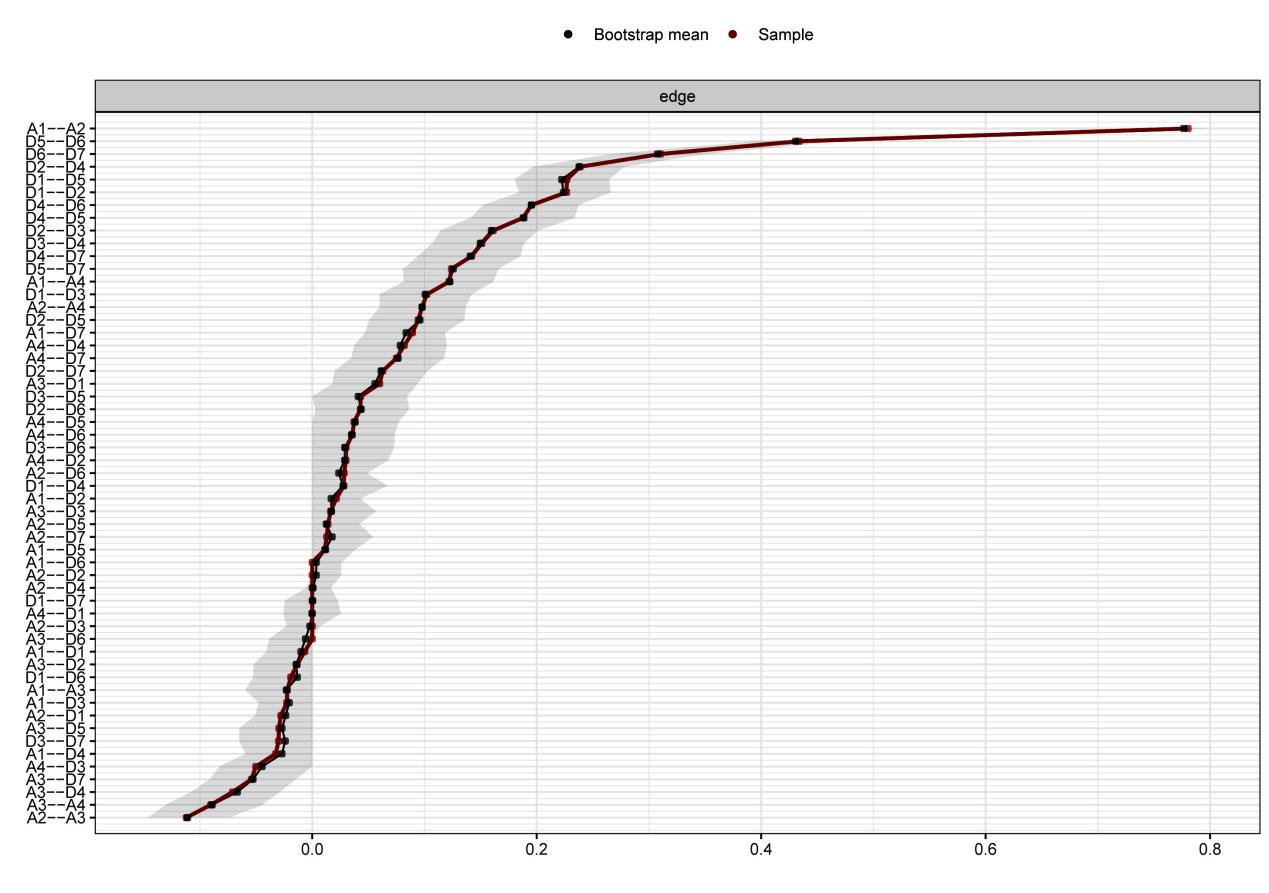
**

**Fig. S1.** Bootstrap Confidence Intervals for Edge Weights in the Total Sample Network

*Note.* In the bootstrap plots, red lines represent the estimated edge weights from the original sample, while gray areas denote the bootstrap confidence intervals; narrower intervals indicate greater precision of estimation.


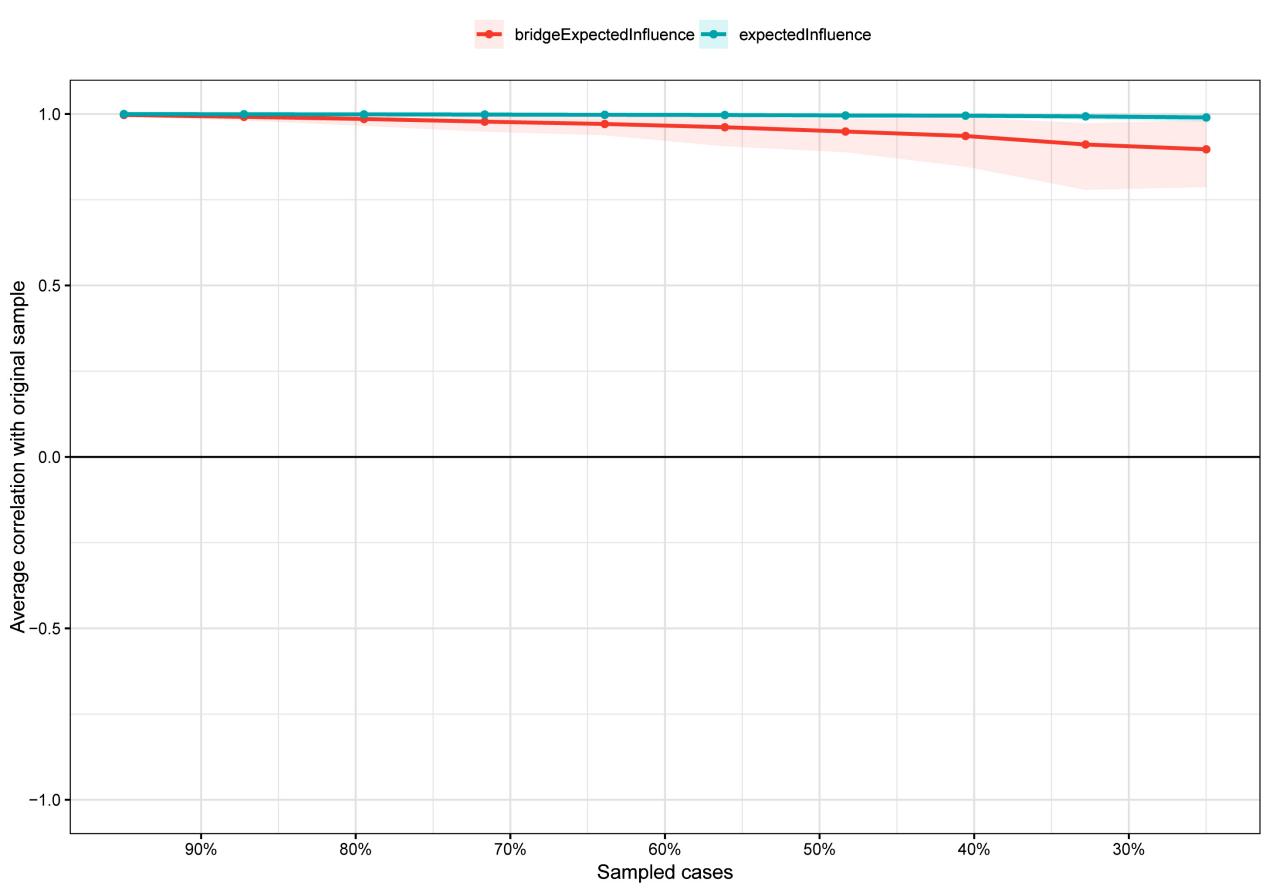


**Fig. S2.** Centrality Stability Analysis Using Case-Dropping Bootstrap in the Total Sample

**
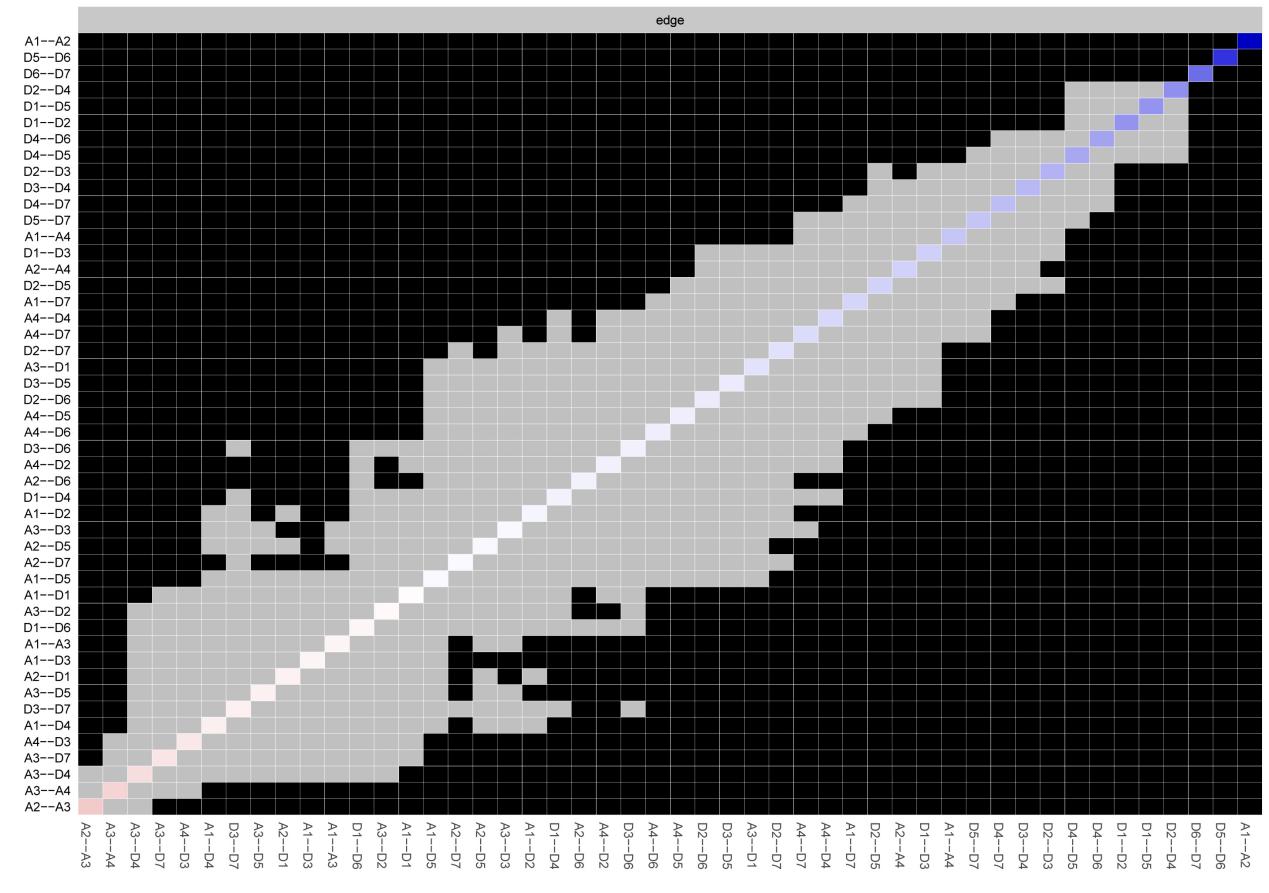
Fig. S3.** Edge Weight Difference Tests in the Total Sample Network


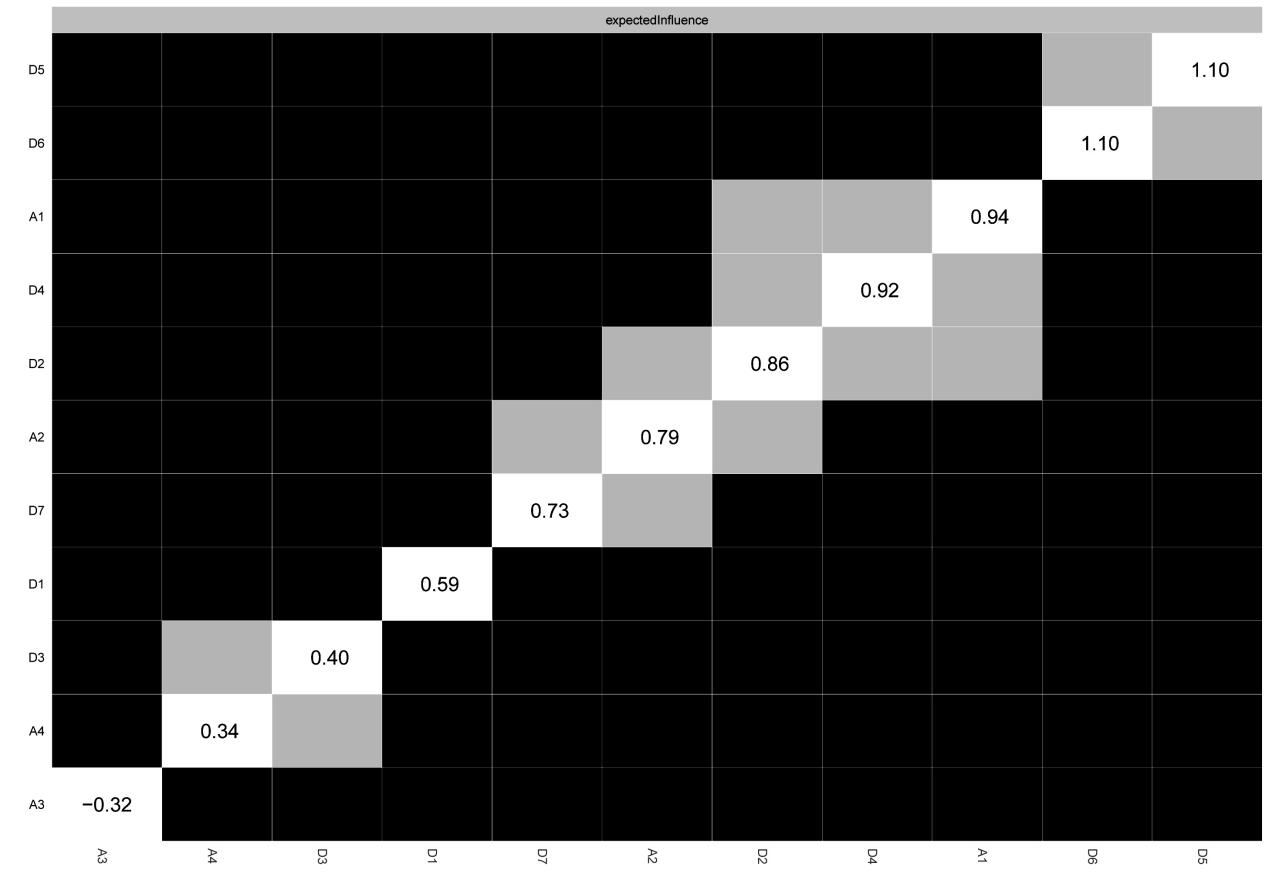


**Fig. S4.** Expected Influence Difference Tests in the Total Sample Network


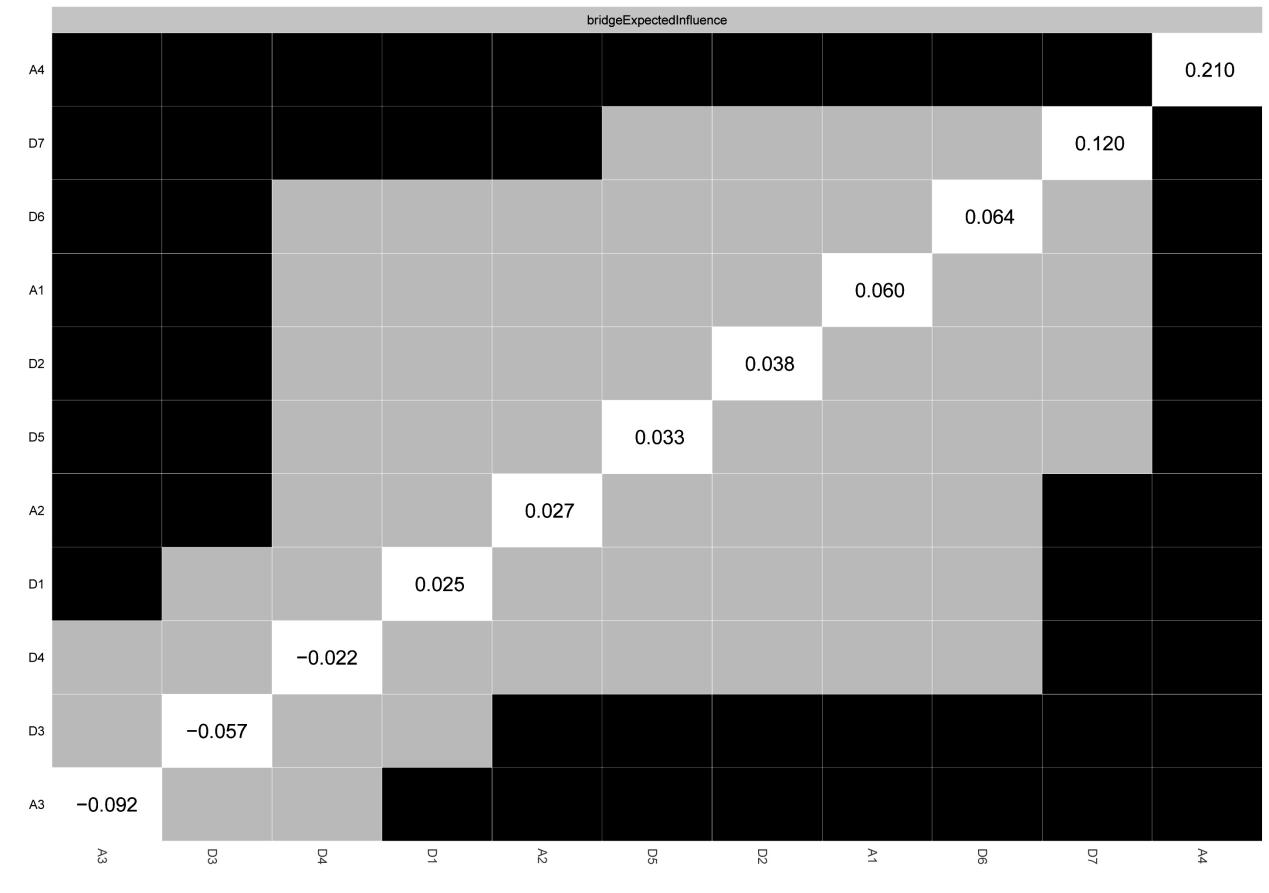


**Fig. S5.** Bridge Expected Influence Difference Tests in the Total Sample Network
